# Reduced m^6^A mRNA methylation is correlated with the progression of human cervical cancer

**DOI:** 10.18632/oncotarget.22041

**Published:** 2017-10-24

**Authors:** Xiuli Wang, Zenghui Li, Beihua Kong, Chen Song, Jianglin Cong, Jianqing Hou, Shaoguang Wang

**Affiliations:** ^1^ Department of Gynecology, Qilu Hospital, Shandong University, Jinan, Shandong 250012, P.R. China; ^2^ The Affiliated Yantai Yuhuangding Hospital of Qingdao University, Yantai, Shandong, 264000, P.R. China; ^3^ Department of Gynecology, Zhucheng Maternal and Child Health Hospital, Zhucheng, Shandong 262200, P.R. China

**Keywords:** m^6^A RNA methylation, cervical cancer, cancer

## Abstract

The m^6^A mRNA methylation involves in mRNA splicing, degradation and translation. Recent studies have revealed that reduced m^6^A mRNA methylation might promote cancer development. However, the role of m^6^A mRNA methylation in cervical cancer development remains unknown. Therefore, we investigated the role of m^6^A methylation in cervical cancer in the current study. We first evaluated the m^6^A mRNA methylation level in 286 pairs of cervical cancer samples and their adjacent normal tissues by dot blot assay. Then the role of m^6^A on patient survival rates and cervical cancer progression were assessed. The m^6^A level was significantly reduced in the cervical cancer when comparing with the adjacent normal tissue. The m^6^A level reduction was significantly correlated with the FIGO stage, tumor size, differentiation, lymph invasion and cancer recurrence. It was also shown to be an independent prognostic indicator of disease-free survival and overall survival for patients with cervical cancer. Reducing m^6^A level via manipulating the m^6^A regulators expression promoted cervical cancer cell proliferation. And increasing m^6^A level significantly suppressed tumor development both *in vitro* and *in vivo*. Our results showed that the reduced m^6^A level is tightly associated with cervical cancer development and m^6^A mRNA methylation might be a potential therapeutic target in cervical cancer.

## INTRODUCTION

Cervical cancer is one of the most prevalent gynecological malignancies in the world [1, 2]. Although major progresses have been made in cancer detection and treatment during the past decades, the 5-year survival rate remains low. Thus, it is important to determine the molecular mechanisms of cervical cancer development, to identify effective prognostic markers and develop novel therapeutic strategies.

Recent studies have uncovered the important role of RNA methylation in cell fate determination, self-renewal and cancer development, indicating a new and promising therapeutic target for investigation [3]. Chemical modified RNA has been demonstrated in recent decades [4]. Among them, the N6-methyladenosine (m^6^A) modification of mRNA/lncRNA is the most prevalent one and plays an important role in gene expression [5, 6].

The m^6^A methylation is reversible and regulated by adenosine methyltransferases [7–9] and demethylases [10, 11]. The m^6^A methyltransferases include METTL3 (Methyltransferase like 3), METTL14 (Methyltransferase like 14), WTAP (Wilms tumor 1-associated protein), RBM15 (RNA binding motif protein 15) and KIAA1429 [7–9, 12]. And the m^6^A demethylases include FTO (Fat mass and obesity-associated protein) and ALKBH5 (AlkB family member 5) [10, 11, 13]. Furthermore, the m^6^A could be selectively recognized by proteins such as HNRNPC (Heterogeneous nuclear ribonucleoprotein C), HNRNPA2B1 (Heterogeneous nuclear ribonucleoprotein A2/B1), YTHDF2 (YTH N6-methyladenosine RNA binding protein 2), YTHDF1 (YTH N6-methyladenosine RNA binding protein 1) and eIF3 (Eukaryotic initiation factor 3) [5, 14–19].

The m^6^A mRNA methylation involves in mRNA splicing, degradation and translation [11, 14, 16–19]. However, its roles in biological processes have just begun to be uncovered [20]. Previous reports have demonstrated that m^6^A mRNA methylation is implicated in stem cell maintenance and differentiation [21–23], and dysregulation of this modification might contribute to development abnormalities, obesity, and other diseases [24, 25]. Recent studies revealed that the m^6^A mRNA methylation also plays an important role in cancer development, such as breast cancer [26, 27], hematologic malignancies [28, 29] and glioblastoma [30, 31]. However, whether m^6^A mRNA methylation also plays an important role in cervical cancer development remains unknown. Therefore, we investigated the role of m^6^A methylation in cervical cancer in the current study.

## RESULTS

### Decreased m^6^A level in cervical cancer biopsies

**Figure 1:**
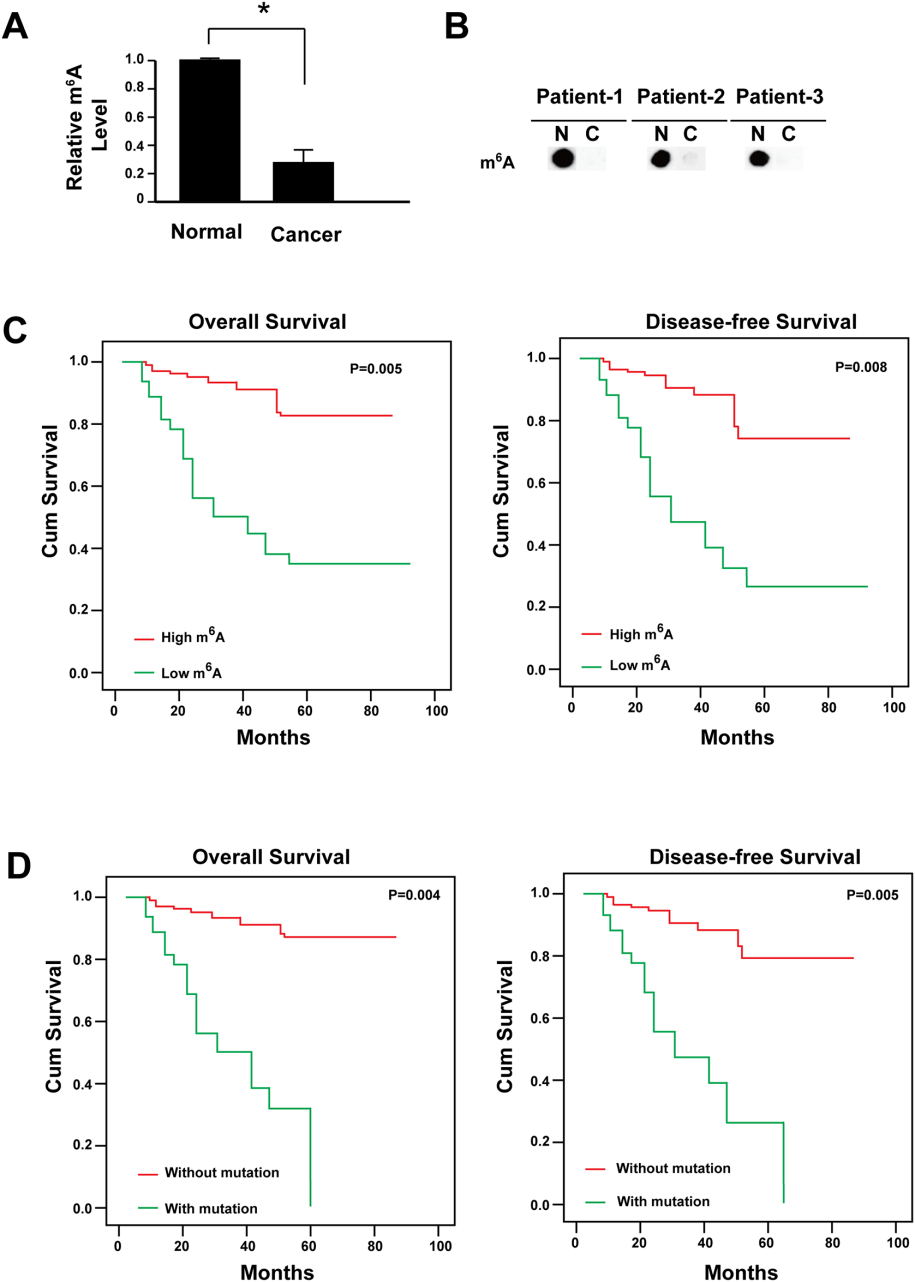
Reduced m^6^A is correlated with the poor prognosis of cervical cancer **(A)** m^6^A levels in cancer tissues and adjacent normal tissues were determined by dot blot (n=286). The relative dot density was measured by Image J. ^*^ *P*<0.05. **(B)** Representative figure for m^6^A levels in cancer tissues and adjacent normal tissues. N: adjacent normal tissues; C: cancer tissues. **(C)** Kaplan-Meier survival curve of patients with high or low level of m^6^A. **(D)** Kaplan-Meier survival curve of patients with or without mutation of m^6^A regulators.

A total of 286 cases with cervical cancer were followed. All these patients had received no pre-operation chemotherapy. They were given the same radical operation and underwent the same adjuvant chemotherapy after the surgery. The m^6^A RNA methylation level was firstly analyzed and the data showed that the m^6^A level reduced significantly in cervical cancer tissues comparing with its paired adjacent non-cancerous tissues (Figure 1A and 1B). Furthermore, the mRNA level of the m^6^A methyltransferases (METTL3 and METTL14) were reduced while the demethylases (FTO and ALKBH5) were increased (Supplementary Figure 1). The reduced m^6^A level was associated with FIGO stage (*P* = 0.008), tumor size (*P* = 0.001), differentiation (*P* = 0.026), lymph invasion (*P* = 0.009) and recurrence (*P* = 0.005, Table 1).

**Table 1:** Association between clinicopathological features and m^6^A mRNA methylation level

	N	m^6^A mRNA methylation level		*P* value
		Low (n=210)	High (n=76)	
Age, years				0.913
<50	214	159	55	
≥50	72	51	21	
FIGO stage				0.008^*^
< IIA	110	58	52	
>IIB	176	152	24	
Tumor size, cm				0.001^*^
<4	124	64	60	
≥4	162	146	16	
Histological type				0.058
Squamous	258	190	60	
others	28	20	8	
Differentiation				0.026^*^
G1 (High)	31	24	7	
G2 (Medium)	122	78	44	
G3 (Low)	133	108	25	
Lymph invasion				0.009^*^
Yes	88	72	16	
No	198	138	60	
Vaginal invasion				
Yes	118	87	31	0.902
No	168	123	45	
Recurrence				0.005^*^
Yes	32	28	4	
No	254	182	72	

^*^P < 0.05 indicates a significant association among the variables.

During the follow-up for all patients, 52 patients had died and 75 experienced recurrence. Disease-free survival (DFS) and overall survival (OS) was conducted to assess the predictive role of m^6^A level for distant metastasis. Both DFS and OS were significantly higher in the high m^6^A level group than the low m^6^A level group (Figure 1C). The low m^6^A level group subsequently developed more recurrence than the high m^6^A level group (*P* < 0.01, Table 1).

It has been demonstrated that the genetic alterations of m^6^A regulators could predict poorer survival in acute myeloid leukemia [29]. Therefore, we also analyzed the gene mutations of m^6^A regulators, including METTL3, METTL14, WTAP, RBM15, KIAA1429, FTO, ALKBH5, HNRNPC, HNRNPA2B1, YTHDF2, YTHDF1 and eIF3 (Supplementary Figure 2). Mutations in individual genes did not show any significant association with clinicopathological features (data no shown). But when all the mutations were pooled together, the m^6^A regulators mutations showed significantly association with FIGO stage (*P* = 0.005), tumor size (*P* = 0.001), differentiation (*P* = 0.016), lymph invasion (*P* = 0.002) and recurrence (*P* = 0.000, Table 2). Both DFS and OS were significantly higher in the patients without m6A regulators mutations (Figure 1D). Univariate and multivariate analysis showed that patients with reduced m^6^A level or containing gene mutations of m^6^A regulators had a significantly reduced OS and DFS (Table 3, Table 4).

**Table 2:** Association between clinicopathological features and m^6^A regulators mutation

	N	m^6^A regulators mutation		*P* value
		No (n=240, %)	Yes (n=46, %)	
Age, years				0.913
<50	214	164	40	
≥50	72	66	6	
FIGO stage				0.005^*^
≤IIA	110	73	37	
≥IIB	176	167	9	
Tumor size, cm				0.001^*^
<4	124	79	45	
≥4	162	161	1	
Histological type				0.168
Squamous	258	213	45	
others	28	27	1	
Differentiation				0.016^*^
G1 (High)	31	30	1	
G2 (Medium)	122	87	35	
G3 (Low)	133	123	10	
Lymph invasion				0.002^*^
Yes	88	87	1	
No	198	153	45	
Vaginal invasion				
Yes	118	102	16	0.912
No	168	138	30	
Recurrence				0.000^*^
Yes	32	32	0	
No	254	208	46	

^*^P < 0.05 indicates a significant association among the variables.

**Table 3:** Univariate Cox proportional hazards model for disease-free survival (DFS) and overall survival (OS)

	DFS			OS		
	HR	95% CI	*P* value	HR	95% CI	*P* value
Age, years						
<50	—			—		
≥50	1.009	0.659–1.841	0.714	0.934	0.530–1.644	0.812
FIGO stage						
≤IIA	—			—		
≥IIB	3.013	0.617–7.662	0.031^*^	3.546	0.860–8.776	0.015^*^
Differentiation						
G1	—			—		
G2	0.816	0.309–2.155	0.681	0.789	0.267–2.334	0.669
G3	1.251	0.718–2.176	0.431	1.145	0.614–2.135	0.671
Tumor size, cm						
<4	—			—		
≥4	5.887	3.025–11.456	<0.001^*^	4.157	2.009–8.602	<0.001^*^
Histological type						
Squamous	—			—		
others	1.108	0.305–4.027	0.877	0.667	0.201–2.215	0.508
Lymph invasion						
No	—			—		
Yes	4.315	0.750–12.306	0.041^*^	4.458	0.764–12.780	0.023^*^
m^6^A level						
Low	4.901	2.469–9.721	<0.001^*^	4.638	2.152–9.997	<0.001^*^
High	—			—		
m^6^A regulator mutation						
No	—			—		
Yes	6.118	3.004–12.462	<0.001^*^	6.348	2.875–14.014	<0.001^*^

^*^P < 0.05 indicates a significant association among the variables.

**Table 4:** Multivariate Cox proportional hazards model for DFS and OS

	DFS			OS		
	HR	95 % CI	*P* value	HR	95 % CI	*P* value
Lymph invasion	2.796	1.919–4.161	<0.001^*^	2.659	1.711–4.223	<0.001^*^
m^6^A level	1.701	1.129–2.541	0.008^*^	3.981	1.854–9.173	<0.001^*^
m^6^A regulator mutation	4.402	1.299–14.551	0.011^*^	8.001	2.403–26.815	<0.001^*^

^*^P < 0.05 indicates a significant association among the variables.

Taken together, the data showed that the reduced m^6^A level might contribute to cervical cancer development and the poor outcomes.

### Reducing m^6^A level promotes cervical cancer cell proliferation

**Figure 2:**
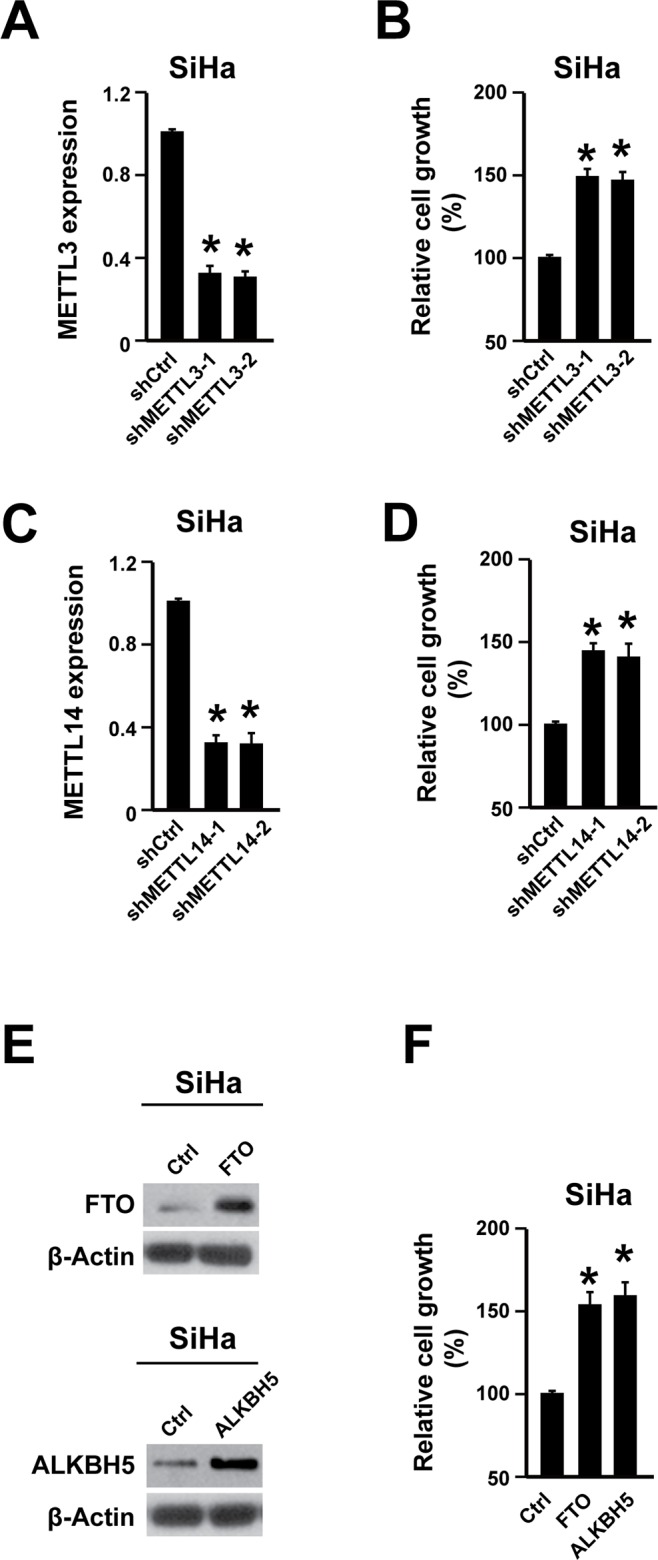
Reducing m^6^A level promoted cervical cancer cell proliferation **(A)** METTL3 shRNA knocking-down efficiency was determined by qPCR in cervical cancer cell line SiHa. n = 3. ^*^ *P*<0.05. **(B)** Knocking down METTL3 promoted cell proliferation. n = 3. ^*^ *P*<0.05. **(C)** METTL14 shRNA knocking-down efficiency was determined by qPCR. n = 3. ^*^ *P*<0.05. **(D)** Knocking down METTL14 promoted cell proliferation. n = 3. ^*^ *P*<0.05. **(E)** Overexpressing FTO and ALKBH5 were validated via western blot. **(F)** Overexpressing FTO and ALKBH5 promoted cell proliferation. n = 3. ^*^ *P*<0.05.

To uncover the important role of m^6^A in cervical cancer development, cell proliferation was analyzed in human cervical cancer cell line SiHa. The m^6^A methylation is regulated by adenosine methyltransferases and demethylases [7–11]. To reduce the m^6^A level, the adenosine methyltransferases (METTL3 and METTL14) were knocked down through two different shRNA. The knock-down efficiency was evaluated with qPCR (Figure 2A-2C, Supplementary Figure 3A). Knocking down both METTL3 and METTL14 promoted SiHa cell proliferation (Figure 2B, 2D). On the other hand, overexpressing adenosine demethylases (FTO and ALKBH5) also decreases m^6^A level. The FTO and ALKBH5 overexpression were validated by western blot (Figure 2E, Supplementary Figure 3B). And their overexpression significantly promoted cell proliferation (Figure 2F). Furthermore, the cell motility was also increased by knocking down METTL3 and METTL14 or overexpressing FTO and ALKBH5 (Supplementary Figure 4).

Thus, reducing m^6^A level could promote cervical cancer cell proliferation, indicating that increasing m^6^A level might have anti-cancer effects in cervical cancer.

### Cervical cancer cell proliferation could be suppressed by increasing m6A level

**Figure 3:**
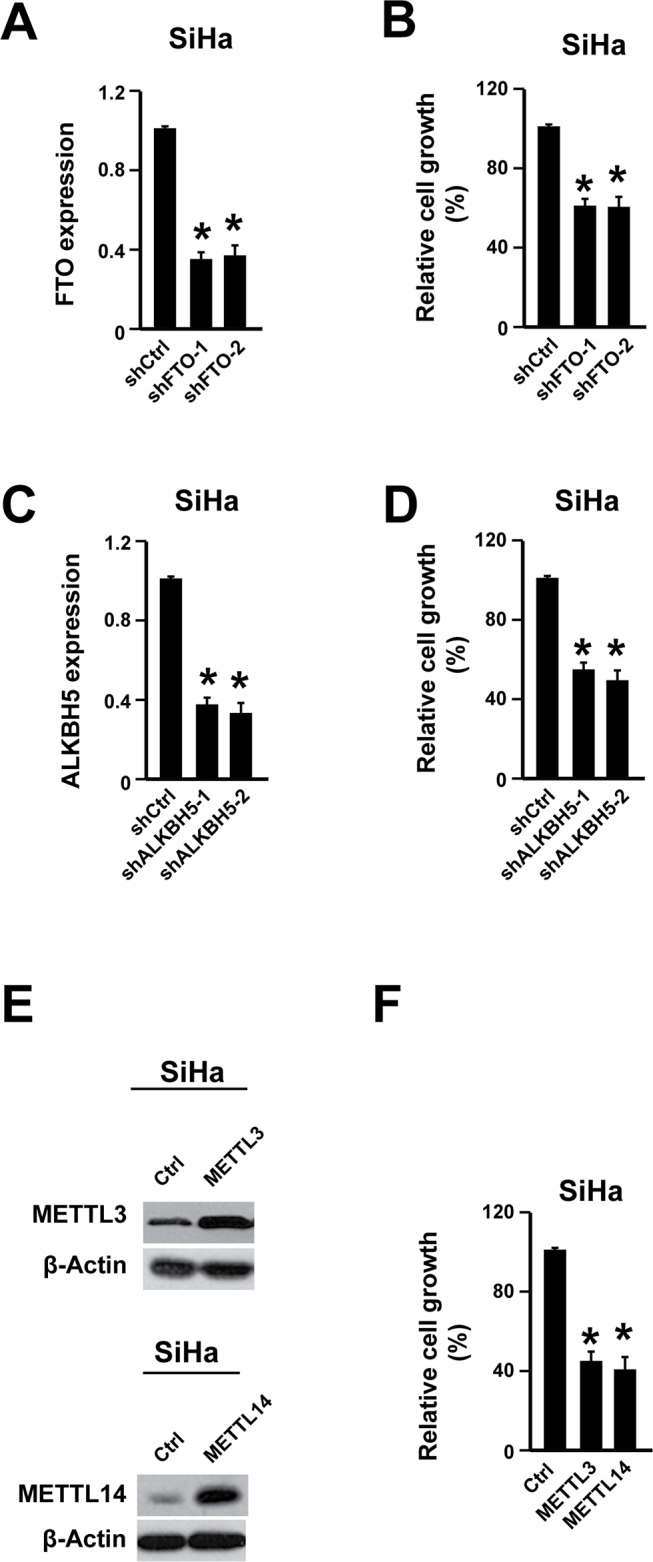
Increasing m6A level suppressed cervical cancer cell proliferation **(A)** FTO shRNA knocking-down efficiency was determined by qPCR in cervical cancer cell line SiHa. n = 3. ^*^ *P*<0.05. **(B)** Knocking down FTO suppressed cell proliferation. n = 3. ^*^ *P*<0.05. **(C)** ALKBH5 shRNA knocking-down efficiency was determined by qPCR. n = 3. ^*^ *P*<0.05. **(D)** Knocking down ALKBH5 suppressed cell proliferation. n = 3. ^*^ *P*<0.05. **(E)** Overexpressing METTL3 and METTL14 was validated via western blot. **(F)** Overexpressing METTL3 and METTL14 suppressed cell proliferation. n = 3. ^*^ *P*<0.05.

As the reduced m^6^A level was tightly associated with poor outcomes and reducing m^6^A in cervical cancer cell lines could promote cell proliferation, we wondering whether increasing m^6^A would have anti anti-cancer effects or not in cervical cancer. Indeed, increasing m^6^A through knocking down adenosine demethylases (FTO and ALKBH5) or overexpressing adenosine methyltransferases (METTL3 and METTL14) suppressed the cancer cell proliferation (Figure 3A-3F, Supplementary Figure 3C-3D). Furthermore, the cell motility was also decreased by knocking down FTO and ALKBH5 or overexpressing METTL3 and METTL14 (Supplementary Figure 5). These data demonstrated that increasing m^6^A might inhibit cervical cancer development.

### Increasing m^6^A level inhibits cervical cancer development *in vivo*

**Figure 4:**
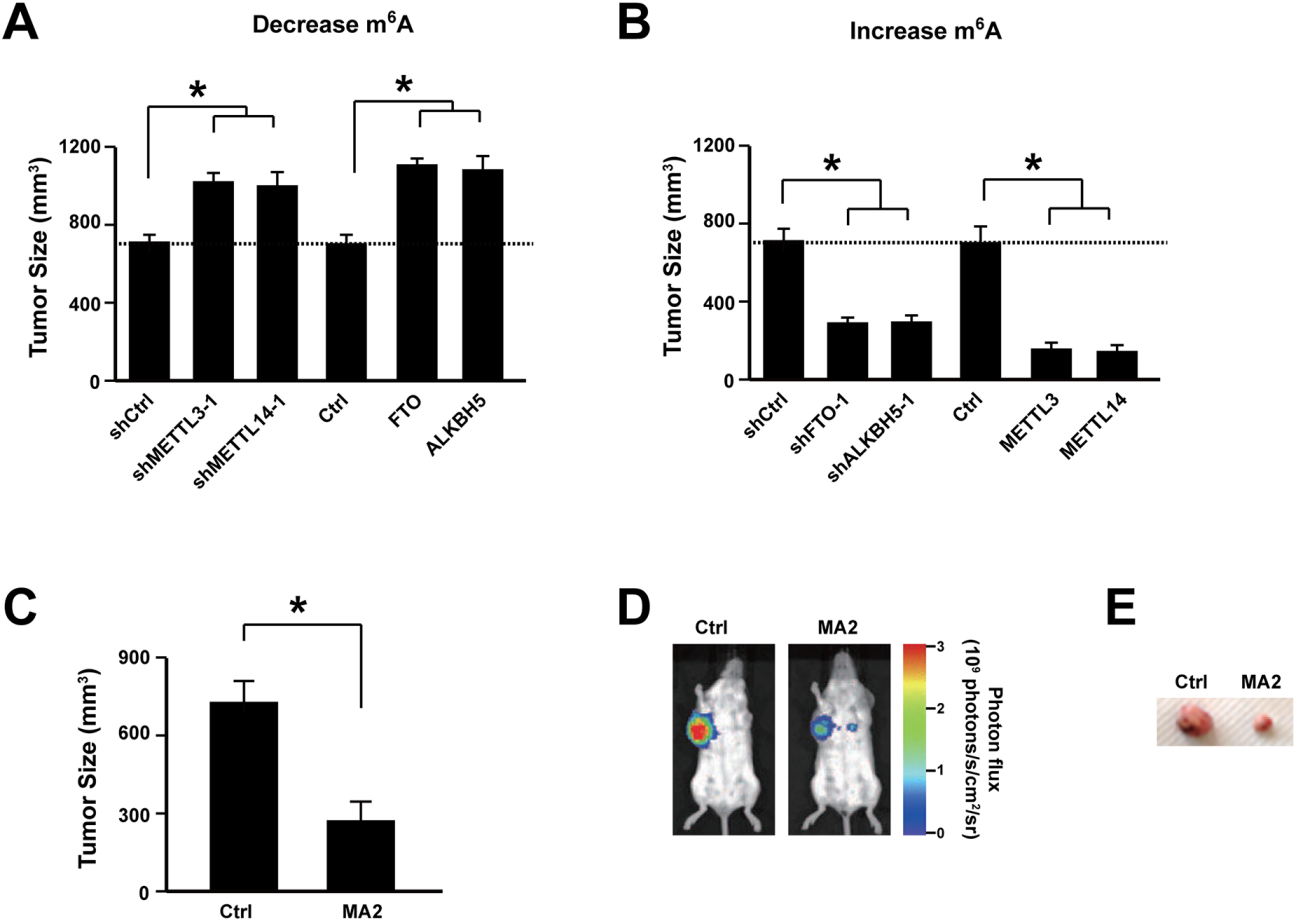
Increasing m6A level suppressed cervical cancer development *in vivo* **(A)** Knocking down METTL3 and METTL14 or overexpressing FTO and ALKBH5 promoted cervical cancer development *in vivo*. n = 12. ^*^ *P*<0.05. **(B)** Knocking down FTO and ALKBH5 or overexpressing METTL3 and METTL14 suppressed cervical cancer development *in vivo*. n = 12. ^*^ *P*<0.05. **(C)** FTO inhibitor MA2 treatment suppressed cervical cancer development *in vivo*. n = 12. ^*^ *P*<0.05. **(D)** Representative luciferase signal density measured via IVIS 50 imaging system. **(E)** Representative tumors isolated from the mice treated with MA2 or control.

To further confirm the important role of m^6^A level on cervical cancer development, human cervical cancer cell line SiHa was first infected with lentivirus expressing shRNA to knock down adenosine methyltransferases (METTL3 and METTL14) or overexpressing adenosine demethylases (FTO and ALKBH5). Then the cells were transplanted subcutaneously into the dorsal scapula region of the NOD/SCID mice. Data showed that decreasing m^6^A level promoted cervical cancer development *in vivo* (Figure 4A). On the other hand, knocking-down adenosine demethylases (FTO and ALKBH5) or overexpressing adenosine methyltransferases (METTL3 and METTL14) suppressed cervical cancer development *in vivo* (Figure 4B). Furthermore, when treating the mice with FTO Inhibitor MA2 [32], the tumor size was significantly reduced (Figure 4C-4E). These data showed that increasing m^6^A level inhibits cervical cancer development *in vivo*.

## DISCUSSION

Despite the current treatments for cervical cancer having improved the survival significantly, the development of drug resistance still occurs in a great number of patients determining recurrence [1, 2]. Therefore, more efforts should be made to uncover the underlying mechanisms and develop novel therapeutic targets.

In the present study, data showed that the m^6^A level was significantly decreased in cervical cancer tissues comparing with tumor paired normal tissues. Further analysis showed that the reduced m^6^A level tightly associated with cancer progression and poor survival, and could be developed as novel prognostic marker to predict tumor recurrence.

Then the m^6^A level was reduced in human cervical cancer cell lines through knocking down the adenosine methyltransferases or overexpressing adenosine demethylases, both of which could promote cell proliferation. On the other hand, increasing m^6^A level suppressed the cancer cell proliferation. The potential therapeutic application of increasing m^6^A was further validated in *vivo*.

In conclusion, the data showed that cervical cancer cells had very low level of m^6^A. Increasing m^6^A level impeded the cervical cancer development. Therefore, m^6^A might be a potential target for cervical cancer treatment.

## MATERIALS AND METHODS

### Patients

A total of 286 pairs of samples were obtained from patients with primary cervical cancer who had undergone surgery without any preoperative therapy at Qilu Hospital of Shandong University between January 2005 and December 2009. Patients staged according to the International Federation of Gynecology and Obstetrics (FIGO) criteria. The histological subtype was assigned according to the criteria of the World Health Organization classification. The clinical and pathologic parameters were reviewed from impatient medical records and presented in Table 1. Samples were collected from the patients consecutively with the standardized protocol. Every patient specimen included two matched pairs, namely, cervical cancer tissues and adjacent normal tissues (≥5 cm away from the tumor). Surgically resected specimens were collected immediately after tumor removal and divided into two aliquots: half were immediately flash-frozen in liquid nitrogen and then frozen at −80°C until RNA and DNA extraction was performed; the remainder was fixed with formalin for histopathological analysis. The diagnosis was confirmed by at least two pathologists. The study was conducted according to the Declaration of Helsinki and approved by the Ethics Committee of Qilu Hospital of Shandong University. Written informed consent was obtained from all patients.

### RNA extraction and m6A dot blot assay

Total RNA was extracted from tissues with Trizol (Invitrogen, Carlsbad, CA, USA) according to the manufacturer's instructions. mRNA was prepared from total RNA using the Dynabeads mRNA purification kit (Ambion, catalog no. 61006). RNA samples were quantified using UV spectrophotometry, and equal amounts were mixed 1:1 with glyoxal loading dye (Ambion) and denatured for 20 min at 50°C. 500ng mRNAs were spotted onto a nylon membrane (GE Healthcare). RNA was UV crosslinked to the membrane, and the membranes were blocked for 1 hr in 5% nonfat dry milk in 0.1% PBST (0.1% Tween-20 in 1x PBS, pH 7.4) (Blocking Buffer). Anti-m^6^A antibody (Synaptic Systems; catalog no. 202003) was diluted 1:1000 in 0.1% PBST and incubated on the membranes for 1 hr (25°C) to overnight (4°C). Following extensive washing with 0.1% PBST, HRP-conjugated donkey anti-rabbit IgG (GE Healthcare) was diluted 1:2500 in Blocking Buffer and added to the membranes for 1 hr at 25°C. Membranes were washed again in 0.1% PBST and developed with enhanced chemiluminescence (ECL; GE Healthcare). The intensity of dot blot signal was quantified by ImageJ.

### Gene mutation analysis

Genomic DNA was extracted from tissues with QIAamp DNA Blood Mini Kit (Qiagen) according to the manufacturer's instructions. Exons were amplified with high fidelity DNA polymerase (NEB) and sequenced with ABI 3730. Sequence deletion, truncation and missense mutations were characterized as gene mutation in the present study.

### Real-time PCR

cDNA was prepared by using the iScript™ cDNA Synthesis kit (Bio-Rad, USA). PCR primers (Generay, Shanghai, China) used for RT-PCR were as follows: for METLL3, sense: 5′-TCCTGACTGACCTTCTTGCTC-3′ and anti-sense: 5′- TCAGCATCGGAA CCAGCAAAG-3′; METLL14, sense: 5′- GTTGGAACA TGGATAGCCGC -3′ and anti-sense: 5′-CAATGCTGTCGGCACTTTCA -3′; FTO, sense: 5′- AACACCAGGCTCTTTACGGTC -3′ and anti-sense: 5′- TGTCCGTTGTAGGATGAACCC -3′; ALKBH5, sense: 5′- ATGCACCCCGGTTGGAAAC -3′ and anti-sense: 5′- GACTTGCGCCAGTAGTTCTCA-3′;β-actin, sense: 5′-CCTGACTGACTACCTCATGAAG-3′ and anti-sense: 5′-GACGTAGCACAGCTTCTCCTTA-3′. RT-PCR amplification reaction was prepared with the SYBR Green PCR kit (Bio-rad, USA) and performed using the 7500 fast Real-Time PCR system (Applied Biosystems, USA). PCR products were verified by melting curve analysis. Relative mRNA levels of target genes were calculated by the 2^-ΔΔct^ method.

### Western blotting

Total protein from cultured cells were lysed in RIPA buffer with protease inhibitor (Beyotime, Shanghai, China). The protein was quantified using a BCA assay kit (Beyotime, Shanghai, China). A total of 20 μg of total protein were separated by 10% SDS-PAGE, transferred onto polyvinylidene fluoride membranes, and then reacted with primary antibodies against METLL3, METLL14, FTO, ALKBH5 and β-actin (all from Abcam, Cambridge, UK). After being extensively washed with PBS containing 0.1 % Triton X-100, the membranes were incubated with alkaline phosphatase-conjugated goat anti-rabbit antibody for 30 min at room temperature. The bands were visualized using 1-step TM NBT/BCIP reagents (Thermo Fisher Scientific, Rockford, IL, USA) and detected by an Alpha Imager (Alpha Innotech, San Leandro, CA, USA).

### Cell culture

The cervical cancer cell lines SiHa was obtained from the American Type Culture Collection (ATCC; Rockville, MD, USA) and cultured in DMEM (GIBCO, Shanghai, China) supplemented with 10 % FBS.

### Plasmid DNA and virus preparation

shRNAs were cloned into lentiviral pLKO.1-puro vector. The following shRNA sequences were used: control shRNA, 5`-ACTCAAAAGGAAGTG ACAAGA-30; METTL3 shRNA-1, 50-GCTGCACTTCAGACG AATT-30; METTL3 shRNA-2, 5`-CCACCTCAGTGGATC TGTT-3`; METTL14 shRNA-1, 5`-GCTAAAGGATGAG TTAAT-3`; METTL14 shRNA-2, 5`-GGACTTGGGATG ATATTAT- 3`; FTO shRNA-1, 5`-GGAAGATTTAAACT CCATGAAG-3`; FTO shRNA-2, 5`-CAAAGTGTTCAA TGGATGCAAC-3`; ALKBH5 shRNA-1, 5`-AGGTTCTC ATATTCTTGGTATC-3`; ALKBH5 shRNA-2, 5`-GATGAAATCACTCACTGCATAC-3`. The METTL3, METTL14, FTO and ALKBH5 expressing lentiviral vector was prepared by cloning the human full coding sequences into the pLVX-puro lentiviral vector.

Lentiviruses were prepared using 293T cells according to the manufacturer's instructions. Cells were incubated with lentivirus and 4 mg/mL polybrene (AmericanBio) for 24 hr.

### Cell growth assay

Cells were seeded at 5×10^4^ cells per well in 24-well plates and cultured for 3 days. Cell number was counted using a hemocytometer.

### Animal study

6–8 weeks old NOD/SCID mice (Charles River Laboratories, Beijing, China) were housed in specific pathogen-free conditions. The study was approved by the Research Ethics Committee of Shandong University. Mice were housed in the pathogen free region and monitored daily during the experiments and the mice would be sacrificed when the weight loss is more than 20%. For evaluation of the tumor growth *in vivo*, 5 × 10^6^ cells were suspended in 200 μl PBS and injected subcutaneously into the dorsal scapula region of the mice. For FTO Inhibitor MA2 treatment, the mice were treated with MA2 (5 nmol in 100μL PBS) or vehicle control by intratumoral injection once a week for 4 weeks. Tumor size was measured with fine digital calipers and calculated by the following formula: tumor volume =0.5 × width^2^ × length. For imaging the tumor clearance *in vivo*, cells were transduced with a lentiviral construct that drives the expression of the click beetle red luciferase (CBR). Bioluminescence imaging was performed 4 weeks after injection. For imaging, mice were injected i.p. with D-luciferin (150 μg/g body weight), anesthetized (2% isoflurane), and placed in an IVIS 50 imaging system (Xenogen). Regions of interest (ROI) were defined manually over the whole body with Living-Image software (Igor Wavemetrics) for determining tumor burden signal intensities.

### Statistical analysis

Data were expressed as mean (±SE) and analyzed by a SPSS software package (SPSS Standard version 13.0, SPSS Inc, USA). Differences between variables were assessed by the Chi-square test. Survival analysis of patients with cervical cancer was calculated by Kaplan-Meier analysis. A log rank test was used to compare different survival curves. A Cox proportional hazards model was used to calculate univariate and multivariate hazard ratios for the variables. Unpaired Student's t test and one way ANOVA were used as appropriate to assess the statistical significant of difference. *P* values under 0.05 were considered statistically significant.

## SUPPLEMENTARY MATERIALS FIGURES


