# Supplementary material for: Reduced m6A mRNA methylation is correlated with the progression of human cervical cancer
Source: Oncotarget. 2017 Oct 24;8(58):98918–30. doi: 10.18632/oncotarget.22041 (PMC5716777; doi:10.18632/oncotarget.22041)
Supplement: Supplementary file 1 [file oncotarget-08-98918-s001.pdf]

Reduced m[6]A mRNA methylation is correlated with the progression of human cervical cancer

SUPPLEMENTARY MATERIALS

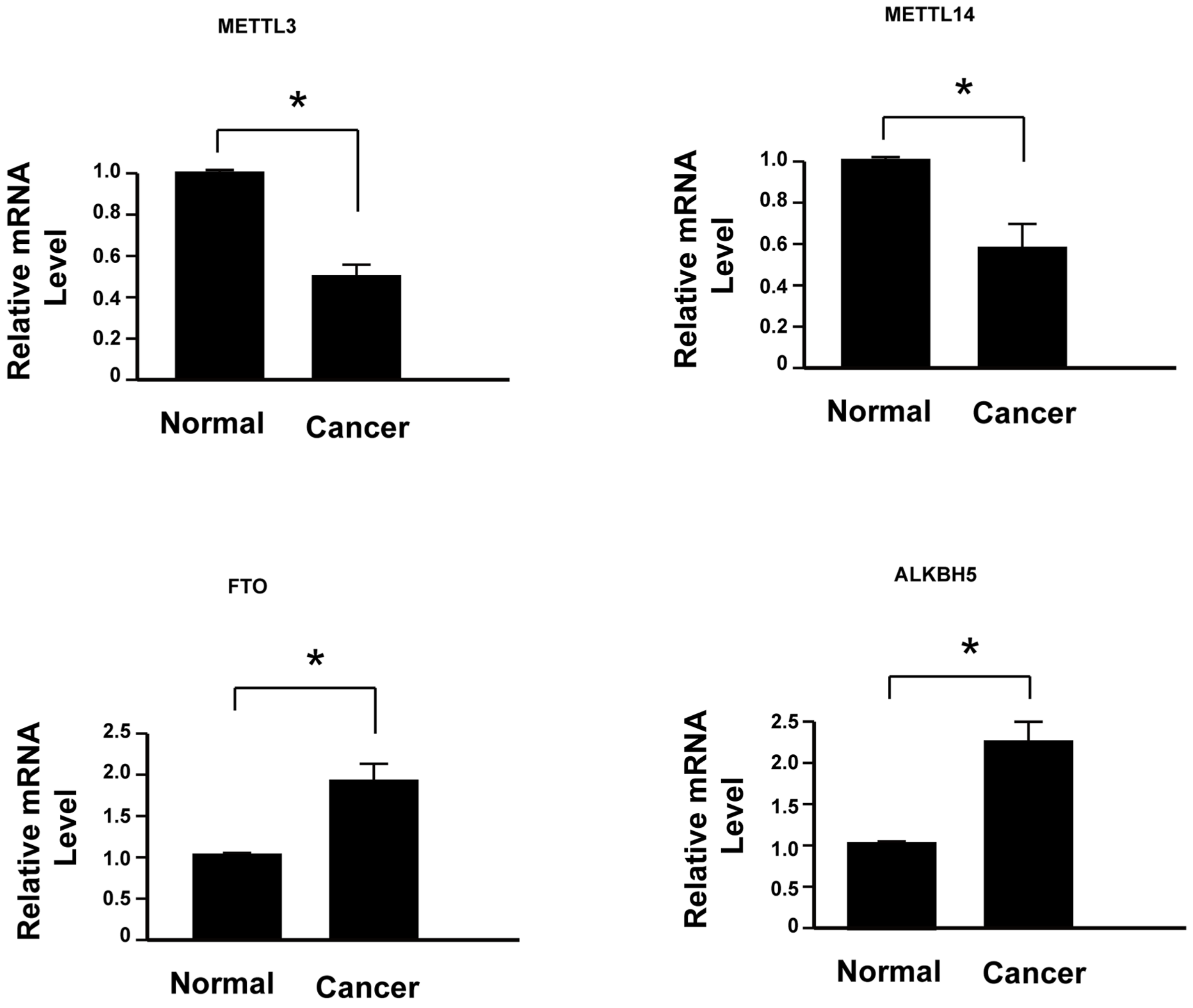

Supplementary Figure 1: The relative mRNA level of METTL3, METTL14, FTO and ALKBH5 was determined by qPCR (n=286). * $P<0.05$.

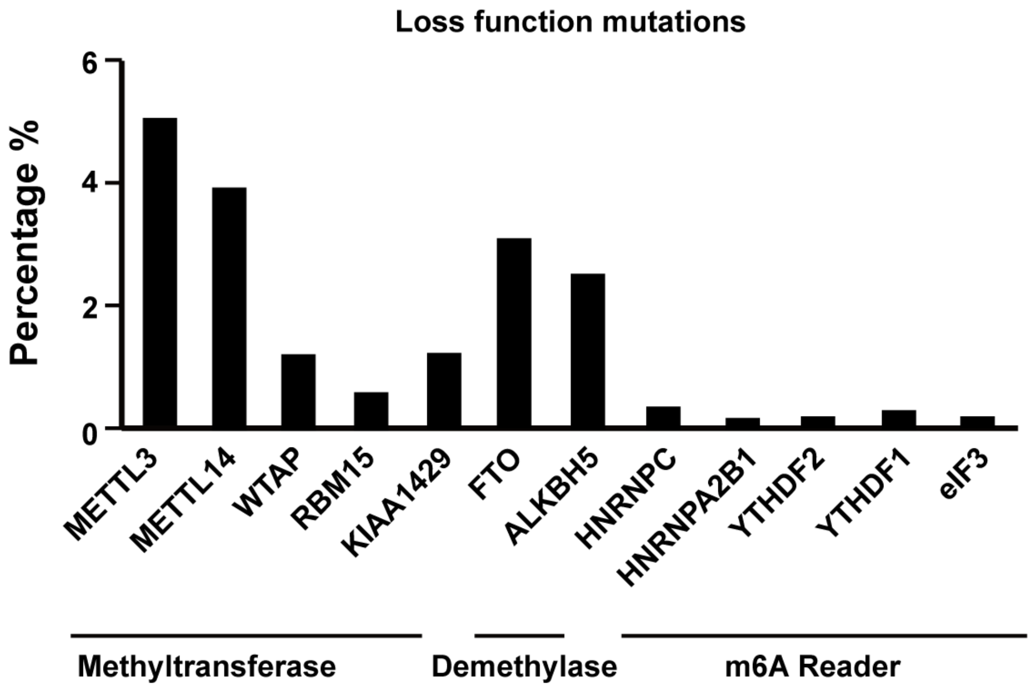

Supplementary Figure 2: Gene mutation frequency that would cause loss function of the gene, including large sequence deletion (>100bp), nonsense mutation, frame shift mutation and missense mutations which are known to cause loss of function (n=286).

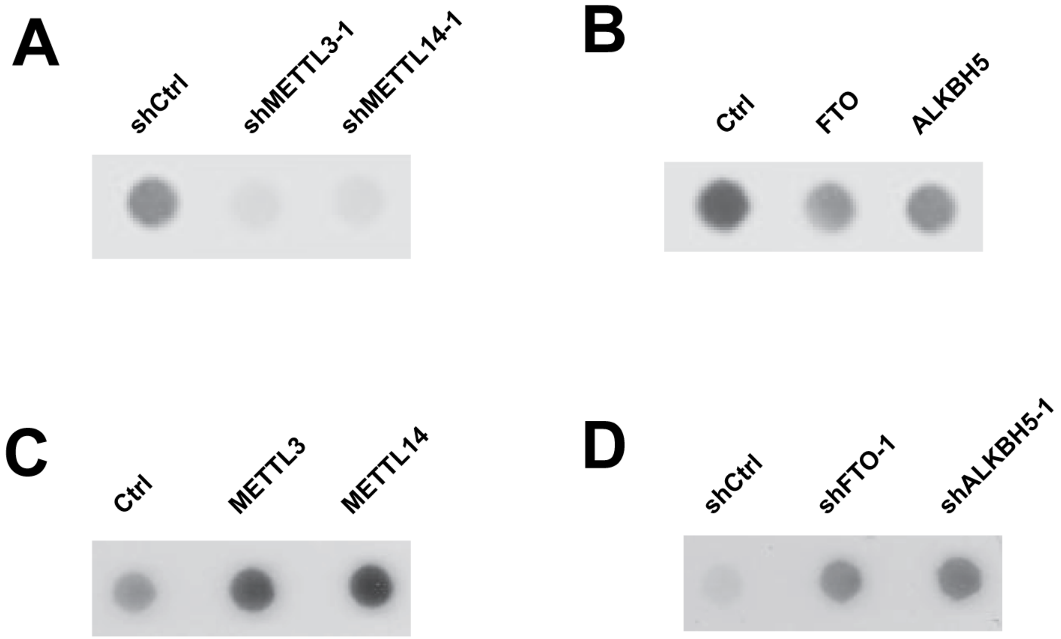

Supplementary Figure 3: Dot blot analysis of m6A levels after knocking down or overexpressing m^{6}A regulations.

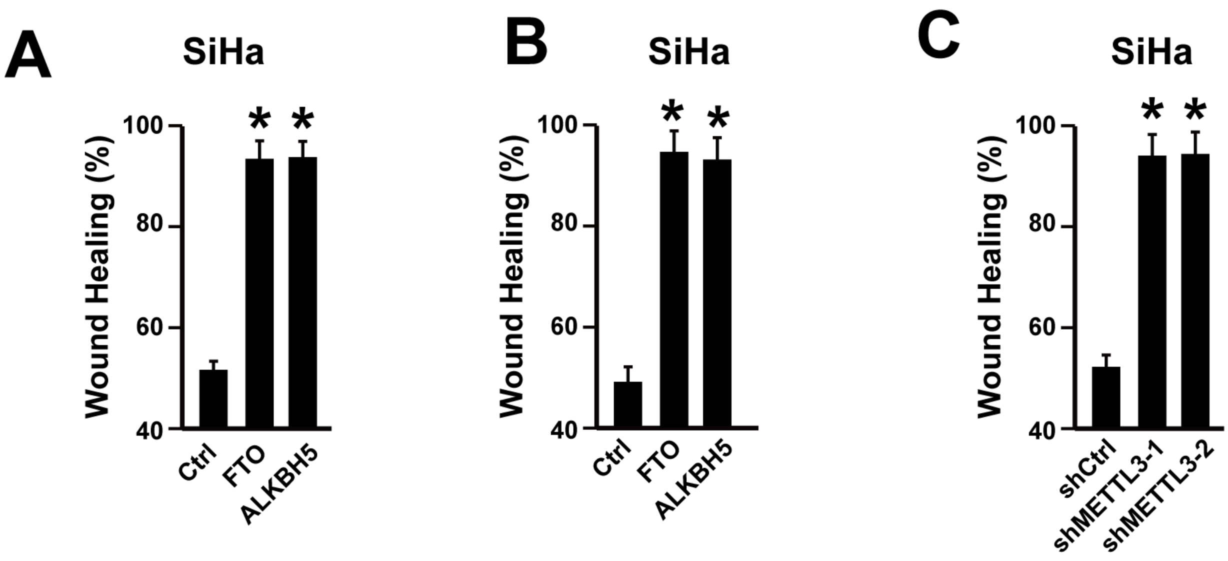

Supplementary Figure 4: Reducing m6A level promoted cervical cancer cell migration. **(A)** Knocking down METTL3 promoted cell migration. n = 3. * P<0.05. **(B)** Knocking down METTL14 promoted cell migration. n = 3. * P<0.05. **(C)** Overexpressing FTO and ALKBH5 promoted cell migration. n = 3. * P<0.05.

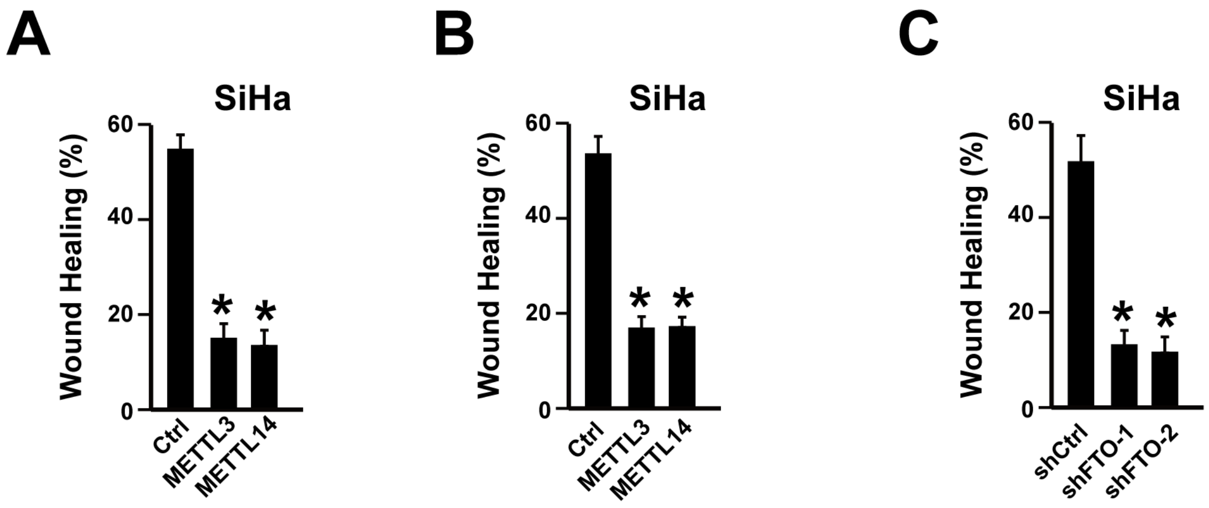

Supplementary Figure 5: Increasing m^{6}A level suppressed cervical cancer cell migration. **(A)** Knocking down FTO suppressed cell migration. n = 3. * *P*<0.05. **(B)** Knocking down ALKBH5 suppressed cell migration. n = 3. * *P*<0.05. **(C)** Overexpressing METTL3 and METTL14 suppressed cell migration. n = 3. * *P*<0.05.
